# 非小细胞肺癌脑转移瘤的治疗进展

**DOI:** 10.3779/j.issn.1009-3419.2012.05.10

**Published:** 2012-05-20

**Authors:** 春华 马, 镕 姜

**Affiliations:** 300060 天津，天津市环湖医院肿瘤介入科 Department of Intervention, Tianjin Huanhu Hospital, Tianjin 300060, China

**Keywords:** 肺肿瘤, 脑转移瘤, 治疗, Lung neoplasms, Brain metastases, Therapy

## Abstract

脑转移瘤是非小细胞肺癌最常见的并发症之一。全脑放疗（whole brain radiotherapy, WBRT）、立体定向放射外科（stereotactic radiosurgery, SRS）、外科手术和化疗是非小细胞肺癌脑转移瘤的重要治疗方式，但其疗效仍不令人满意，而综合治疗能有效地延长患者生存期、改善生活质量。近年来随着治疗技术的进步，分子靶向治疗和患者的生存期及生活质量越来越受到重视。本文就非小细胞肺癌脑转移瘤的治疗现状和综合治疗的进展做一综述。

## Abstract (en)

Brain metastases is one of the most common complications of non-small cell lung cancer, whole brain radiotherapy (WBRT), stereotactic radiosurgery (SRS), surgery and chemotherapy are standard methods in the treatment of brain metastases. But the effect of those treatments are still sad. Comprehensive treatment can prolong the survival and improve the quality of life. Recently, the improvement of technology, targeted therapy, survival time and the quality of life are in increasingly concerned. The paper make a summary of current situation and progress for comprehensive therapy of brain metastases.

肺癌是我国目前最常见的恶性肿瘤之一，其发病率和病死率呈逐年上升趋势，非小细胞肺癌（non-small cell lung cancer, NSCLC）约占所有肺癌的80%，中晚期NSCLC的放射及化学治疗有效率仅为20%-40%，5年生存率8%-12%^[1]^。肺癌患者预后不良的主要原因是局部复发或远处转移，其中脑转移（brain metastases, BM）发生率达30%-50%^[2, 3]^，以腺癌最多见，患者预后极差，自然中位生存期仅1个月-2个月^[4]^。脑转移瘤的治疗方法主要有全脑放疗、立体定向放射外科、外科手术和化疗，但单一治疗方式的疗效并未获得突破性进展^[5-7]^。随着对NSCLC脑转移瘤研究的不断深入、治疗技术的进步及对患者生活质量（quality of life, QoL）的重视，制定综合治疗方案已成为个体化治疗的热点，本文就此做一综述。

## 1 放射治疗

### 1.1 全脑放射治疗

全脑放射治疗（whole brain radiotherapy, WBRT）是NSCLC脑转移瘤的标准治疗方式之一，尤其适合一般状态较差、高龄、KPS评分较低及颅内多发转移灶的患者。射线种类一般采用60Co-γ线或者加速器的6 MV-X线，方法为全脑两侧野对穿照射，肿瘤剂量一般为3, 000 cGy-4, 000 cGy/2 w-4 w。WBRT可有效地改善患者的神经症状和功能、改善生活质量，但由于晚期肿瘤患者对治疗的耐受性差、肿瘤复发及原发病灶未控制，治疗总有效率为60%-80%，患者中位生存期为3个月-6个月^[8, 9]^。糖皮质激素及高效放疗增敏剂的联合应用，可增加WBRT的短期疗效。WBRT主要毒副反应是加重或促进脑细胞水肿，致使颅内压增高，长期存活的患者可能会出现迟发性脑神经细胞的损伤，多表现为顽固性头痛、智力下降、认知功能障碍等。由于全脑放疗的毒副反应及对患者生活质量的负面影响，且预防性全脑照射（prophylactic cranial irradiation, PCI）仅能有效降低局部晚期NSCLC（locally advanced NSCLC, LA-NSCLC）脑转移的发生率，对患者的总生存期（overall survival, OS）及无疾病进展时间（progression free survival, PFS）无明显改善，PCI的适应人群及介入时间仍存在着争议^[10-12]^。

### 1.2 立体定向放射外科（stereotactic radiosurgery, SRS）

立体定向放射外科是采用立体定向旋转聚焦的方法，一次性地将分散的高能X射线或γ射线聚集到靶点，在肿瘤周边形成了一个非常陡峭且边缘锐利的剂量梯度分布，对肿瘤的毁损形如刀切一般，故又称为X-刀和γ-刀^[6]^。SRS分割模式为单次大剂量，多为10 Gy-30 Gy，主要根据肿瘤的直径和周围重要器官的耐受量决定。若分割模式为多次方案称为立体定向放疗（stereotactic radiotherapy, SRT）。SRS和SRT优点是定位准确，靶体积内外剂量落差大，可有效地杀灭肿瘤组织而最大程度地保护周围正常的脑组织，使瘤体迅速消退，且毒副反应小。放射外科(X-刀、γ-刀)已成为治疗NSCLC脑转移的有效治疗方法之一，治疗总有效率达80%-90%，中位OS为9个月-10个月。Motta等^[13]^研究373例γ-刀治疗NSCLC脑转移，结果显示患者中位OS为14.2个月；Mariya等^[14]^研究28例多次重复SRS治疗NSCLC脑转移，结果显示患者2年和4年生存率分别为51%和23%，中位OS高达26个月。上述研究结果均显示出SRS较好的重复性及肿瘤的控制率。然而SRS受限于肿瘤直径（< 3 cm）、转移瘤数目（< 5个）、转移瘤位置（位置较深或位于重要功能区）、不能取得病理诊断、患者全身情况以及治疗后较严重的瘤周水肿、正常脑组织坏死等因素，其仍不能完全替代WBRT和外科手术。

### 1.3 全脑放疗联合立体定向放射外科（WBRT+SRS）

WBRT照射范围广，能较好的控制颅内多发转移瘤的生长，但放射总剂量相对受限，易导致肿瘤控制不理想。SRS将高能射线聚焦照射，使受照射肿瘤病灶获得较好的控制，但对肿瘤边缘及多发转移灶控制欠佳，易出现复发。二者联合可以优势互补，提高临床疗效、改善患者生活质量。Minniti等^[15]^研究66例WBRT联合SRS治疗NSCLC脑转移，结果显示联合治疗组中位OS为10.3个月，单纯WBRT组中位OS为7.2个月（*P*=0.005），联合治疗组6个月及12个月疾病控制率分别为82%和42%。WBRT联合SRT的治疗方式尤其适合颅内肿瘤 > 3个的患者，相对单纯WBRT能明显延长患者生存期，提高疾病控制率。

## 2 外科手术治疗

外科手术治疗是治疗NSCLC脑转移瘤的方法之一，手术切除颅内转移瘤可以迅速解除或减轻患者的颅内高压，改善临床症状，延长生存期，提高生活质量，并能取得病理诊断。目前认为NSCLC脑转移瘤的手术适应征为^[16]^：①原发病灶已切除或行放化疗，局部无复发；②肿瘤位置表浅或位于非重要功能区；③预计术后患者生存3个月以上；④患者卡氏评分较高，可耐受手术；⑤对肿瘤位于重要功能区无法手术切除的患者，可行减压手术+活检，术后行辅助放化疗；⑥病灶位于小脑，因其易影响脑脊液循环导致脑疝，宜积极手术。随着外科技术的进步及术中定位技术的发展，很大程度上提高了手术疗效，降低了手术风险，但单纯手术治疗仍面临术后残存肿瘤复发、新发转移灶及不能控制原发肿瘤进展等问题。国内外研究资料均支持手术联合WBRT、SRS或者化疗的治疗方式。

## 3 化疗

近年研究^[17]^表明，在肿瘤脑转移的过程中血脑屏障（blood brain barrier, BBB）已遭到破坏，且全脑放疗、甘露醇等脱水药物亦可使BBB不同程度地开放，因此化疗药物可以顺利地通过血-脑脊液屏障，进入中枢神经系统杀伤肿瘤细胞。对NSCLC脑转移瘤效果较好的药物有亚硝脲类药物如尼莫司汀（ACNU）和洛莫司汀（CCNU）以及其它如顺铂（DDP）、替尼泊苷（VM-26）、替莫唑胺（TMZ）、紫杉醇等。顺铂联合其它化疗药物是治疗NSCLC脑转移的常用方案，患者中位OS为6个-9个月^[18, 19]^，Barlesi等^[20]^报道了43例使用培美曲塞联合顺铂一线化疗治疗NSCLC脑转移，总有效率为34.9%，中位OS为7.4个月。马春华等^[21]^报道27例使用替尼泊甘、尼莫司汀联合卡铂动脉灌注化疗治疗NSCLC脑转移，总有效率为55.56%（15/27），中位OS为7个月。由于NSCLC脑转移患者化疗耐受能力差、颅内病情进展和多次化疗后对化疗药物耐药等问题，单纯化疗疗效仍不令人满意^[22]^。

## 4 分子靶向药物治疗（molecular targeted therapy, MTT）

近年来，针对细胞受体、基因、调控分子等信号传导为目标的分子靶向药物治疗NSCLC逐渐受到重视。研究^[23]^证实表皮生长因子受体（epidermal growth factor receptor, EGFR）在45%-70%的NSCLC中呈过度表达。EGFR的酪氨酸激酶抑制剂（tyrosine kinase inhibitor, TKI）在NSCLC治疗中取得较好的疗效，肺癌患者对EGFR-TKI的敏感性与*EGFR*基因突变密切相关^[24]^。国外文献报道^[25, 26]^NSCLC脑转移患者*EGFR*基因突变率高达44%-50%，提示NSCLC脑转移患者接受厄洛替尼治疗疗效良好。目前治疗NSCLC常用的EGFR-TKI类药物主要有吉非替尼（gefitinib，易瑞沙）和厄洛替尼（erlotinib，特罗凯）。吉非替尼是一种高选择性EGFR-TKI，能竞争性地结合ATP、抑制EGFR细胞内的酪氨酸激酶区域的自磷酸化，阻断下游信号的传递，从而抑制肿瘤新生血管生成、细胞增殖、侵袭及转移。厄洛替尼是一种新型的奎纳唑啉类化合物，它能抑制EGFR酪氨酸激酶的自磷酸化反应，还可诱导细胞周期抑制蛋白P27的表达，使癌细胞阻滞于G_1_期，从而达到抑制癌细胞增殖的作用。白皓等^[27]^报道50例使用吉非替尼治疗NSCLC脑转移患者，疾病控制率为84%，中位PFS为7.0个月，中位OS为10.8个月。汤传昊等^[28]^报道104例使用厄洛替尼治疗化疗失败的晚期NSCLC患者，其亚组分析21例脑转移瘤患者显示，疾病控制率为81%，中位PFS为6.1个月，中位OS为12.1个月。2011年，世界肺癌大会（WCLC）上公布一项厄洛替尼二线治疗晚期无症状脑转移NSCLC患者的Ⅱ期研究（CTONG0803），结果显示总有效率（overall response rate, ORR）达56.3%，中位PFS为10.1个月，6个月生存率为87%，1年生存率为74%。上述研究均提示EGFR-TKI治疗NSCLC脑转移能提高患者生活质量，延长生存期。由于EGFR-TKI治疗的敏感性与EGFR基因是否突变密切相关，对于亚裔、女性、不吸烟、肺腺癌的患者疗效确切^[29, 30]^，EGFR-TKI仍用于NSCLC脑转移的二线或三线治疗方案。

## 5 综合治疗

目前，NSCLC脑转移的传统治疗方式都是姑息性的，且每种治疗方式都有其优缺点。随着对NSCLC脑转移瘤研究的不断深入、治疗技术的进步及对患者生活质量的重视，针对患者生活状态评分、神经功能状态、原发肿瘤和脑转移瘤的部位、病理类型、大小和数目，有无颅外转移等因素，制定综合治疗方案已成为个体化治疗的热点。Pesce等^[31]^报道59例采用全脑放疗联合吉非替尼或替莫唑胺治疗NSCLC脑转移患者的研究，结果显示联合吉非替尼组患者中位OS为6.3个月，联合替莫唑胺组患者中位OS为4.9个月，全组患者对治疗的耐受性良好，无不良反应。Cortot等^[32]^报道50例使用替莫唑胺、顺铂联合全脑放疗治疗NSCLC脑转移患者的Ⅱ期临床研究，结果显示中位PFS为2.3个月，中位OS为5个月。2011年，美国临床肿瘤学年会（ASCO）上公布一项采用厄洛替尼联合全脑放疗治疗伴脑转移NSCLC的Ⅱ期研究^[26]^，结果显示厄洛替尼联合WBRT可明显延长中位PFS至10.9个月。白皓等^[33]^报道了352例肺癌脑转移患者治疗及预后分析，其中对症治疗组28例、单纯放化疗组49例、放疗联合化疗组192例、立体定向放射治疗（γ-刀）联合化疗或放化疗组72例和手术联合化疗或放化疗组11例，结果显示患者中位OS分别1.7个月、3.2个月、9.0个月、11.6个月、17.1个月。上述结果均支持综合治疗的个体化治疗方案是治疗肺癌脑转移的主要研究方向。

## 6 结论

综上所述，针对NSCLC脑转移应联合多种治疗方式的综合治疗，并且根据患者的具体情况制定出个体化的治疗方案。放射治疗引起放射性脑损伤的预防和治疗、PCI介入时间及适应人群、立体定向放射外科的剂量分割模式、分子靶向药物的应用范围探索、外科手术后的联合治疗方案及对患者生活质量的影响等问题，仍是我们今后研究的主要方向。只有多学科的综合治疗、优势互补，才能达到提高临床疗效，改善患者生活质量和延长生存期的最优治疗效果。
